# Prevalence of Serum Antibody Titers against Core Vaccine Antigens in Italian Dogs

**DOI:** 10.3390/life13020587

**Published:** 2023-02-20

**Authors:** Paola Dall’Ara, Stefania Lauzi, Jari Zambarbieri, Francesco Servida, Laura Barbieri, Roberto Rosenthal, Lauretta Turin, Elena Scarparo, Joel Filipe

**Affiliations:** 1 Department of Veterinary Medicine and Animal Sciences (DIVAS), University of Milan, Via dell’Università 6, 26900 Lodi, LO, Italy; 2 Clinica Veterinaria Pegaso, Via Dante Alighieri 169, 22070 Rovello Porro, CO, Italy; 3 Clinica Veterinaria Turro, Via Gerolamo Rovetta 8, 20127 Milano, MI, Italy; 4 Ambulatorio Veterinario di Favignana, Via Daniele Manin, 10, 91023 Favignana, TP, Italy

**Keywords:** dog, core vaccinations, canine parvovirus type 2 (CPV-2), canine distemper virus (CDV), canine adenovirus type 1 (CAdV-1), antibody titration, VacciCheck

## Abstract

Canine vaccination is the main tool for preventing dangerous and widespread diseases. The strongly recommended (core) dog vaccines are against Canine Parvovirus type 2 (CPV-2), Canine Distemper Virus (CDV), and Canine Adenovirus (CAdV-1), but vaccination protocols should be tailored to dog lifestyles. Vaccination guidelines suggest vaccinating adult dogs no more frequently than every 3 years using modified live (attenuated) vaccines (MLV), thus obtaining a long-lasting (sometimes throughout life) specific protection in many but not all animals. The aim of this study was to determine the actual levels of seroprotection against CPV-2, CDV and CAdV-1 in a cohort of Italian dogs by using the in-practice test VacciCheck. A total of 1,027 dogs (951 vaccinated and 76 unvaccinated) were analyzed for Protective Antibody Titers (PATs) against CPV-2, CDV, and CAdV-1. Differences related to sex, age, breed size, health status, and time elapsed since last vaccination were evaluated. Half of the entire canine cohort (50.6%) had PATs for all three viruses (68.5% considering only vaccinated dogs). In particular, 90.8% of dogs were protected against CPV-2, 68.6% against CDV, and 79.8% against CAdV-1. Most dogs remained protected for 3 years after vaccination or longer. Revaccination on a 3-year basis can then be recommended for core MLV vaccines without altering individual’s seroprotection or even herd immunity.

## 1. Introduction

Vaccination is the main tool for preventing dangerous and widespread diseases both in humans and in veterinary practice. WSAVA (World Small Animal Veterinary Association [1]) and other international vaccination guidelines (American Animal Hospital Association—AAHA [2], Australian Veterinary Association—AVA [3], British Veterinary Association—BVA [4], Canadian Veterinary Medical Association—CVMA [5,6]), some of which are specific only to cats [7,8,9,10,11], classify pet vaccines as core and non-core ones. Core vaccines are intended for all dogs since they protect against terrible hazardous and life-threatening diseases, while non-core ones are optional and recommended only for animals at risk of contracting specific infections.

Parvovirus infection is a contagious and often fatal disease in dogs, caused by Canine Parvovirus type 2 (CPV-2), a small naked DNA virus belonging to the family *Parvoviridae*, subfamily *Parvovirinae*, genus *Parvovirus*, and species *Carnivore protoparvovirus 1*. Over the years, the circulation of CPV-2 has been increasingly waning until its disappearance, giving way to three antigenic variants (2a, 2b, 2c) variously distributed worldwide. The infection causes severe hemorrhagic gastroenteritis accompanied by lymphopenia especially in puppies and in unvaccinated dogs and is characterized by high morbidity and mortality despite the availability of excellent vaccines [12,13,14,15,16,17,18]. Distemper is a worldwide diffuse and extremely contagious disease caused by Canine Distemper Virus (CDV), a single-stranded RNA enveloped virus, belonging to the *Paramyxoviridae* family, genus *Morbillivirus*. The virus infects dogs and many other domestic and wild carnivores and causes severe immunosuppression with multisystem involvement (respiratory, gastrointestinal, skin, central nervous system), culminating in neurological involvement and progressive demyelination [17,19,20,21]. Infectious canine hepatitis (Rubarth’s hepatitis) is caused by Canine Mastadenovirus A (CAdV-1), a double-stranded naked DNA virus belonging to the family *Adenoviridae*, genus *Mastadenovirus*. The infection mainly affects dogs and other carnivores and damages the kidneys, liver, and eyes. Since the middle of the last century, its incidence in the canine population has been drastically reduced worldwide, thanks to systematic vaccination [17,22,23,24,25].

Core vaccines are designed for these three highly contagious, widespread, and very often fatal diseases and are therefore strongly recommended. All dogs should be vaccinated with core vaccines at least once in their life for a dual purpose: to prevent individual infections and to assure herd immunity [1,2,26]. For decades, vaccination for these diseases was traditionally performed on an annual basis. Nowadays, due to new knowledge and modern vaccines availability, all the international guidelines on good vaccination practices along with many immunologists and vaccine experts suggest vaccinating adult dogs no more frequently than every 3 years using modified live (attenuated) vaccines (MLV) [1,2,26,27,28]. Even this practice, however, is a fair “trade-off”, since MLV vaccines confer protection for a time span considerably longer than 3 years. Many studies have indeed demonstrated the persistence of specific vaccine-induced protection for many years (sometimes throughout life) after vaccination in many but not all animals [28,29,30,31]. Several factors can in fact interfere with the mounting of an adequate immune protection, firstly the interference of Maternally Derived Antibodies (MDA) in puppies [14,31,32,33,34]. Therefore, exactly knowing the puppy’s antibody protection would help to reduce both vaccination failures and unnecessary vaccinations. Moreover, antibody titration could also help in identifying older dogs that are no longer protected [35,36,37,38,39], or the so-called non-responder dogs, which are genetically unable to mount a protective immunity against either field or vaccine pathogens. The literature reports that 1 out of 1000 dogs may be a non-responder to CPV-2, 1 out of 5,000 to CDV, and only 1 out of 100,000 to CAdV-1 [1,14,26,28].

For all these different reasons, current position statements from different guidelines and experts emphasize that vaccination should be individualized and tailored to pet lifestyles. In this regard, assessing the actual immune status of each dog (and cat) using simple in-practice kits is gaining momentum in the world of pet veterinarians [1,29,40].

The aim of this study was to determine the actual levels of seroprotection against CPV-2, CDV, and CAdV-1 in a large cohort of Italian dogs by using an in-practice test kit.

## 2. Materials and Methods

### 2.1. Study Population and Study Protocol

Serum/plasma samples used for this study were collected over 9 years (January 2014 to December 2022) both to analyze antibody titer and for other purposes. According to the Ethical Committee decision of the University of Milan, residual aliquots of samples collected under informed consent of the owners can be used for research purposes without any additional formal request of authorization (EC decision 29 October 2012, renewed with the protocol n. 02-2016). For each dog, key information was recorded: (1) sex and reproductive status: intact or neutered female or male; (2) age: puppies and juveniles (simplified puppies) from 16 weeks of age (after the end of the eventual first vaccinations series) to less than 1 year of age, adults, seniors, and geriatrics (for these last three—adult, senior, and geriatric—the age category was determined based on canine size according to the rule that small dogs live longer than large ones and vice versa [41,42]); (3) breed (purebred or crossbred) and breed size: small (<10 kg), medium (≥10–<25 kg), and large (≥25 kg); (4) health status: healthy or unhealthy (only those clinical problems that could impact immune function were considered; see Results section); (5) vaccination history, considering time elapsed since last vaccination: ≤1 year, >1 year–≤3 years, or >3 years.

### 2.2. Detection of Specific Antibodies by VacciCheck

Each serum/plasma sample was assayed using the in-clinic test Canine VacciCheck (produced in Israel by Biogal, Kibbutz Galed, Israel, and supplied in Italy by Agrolabo, Scarmagno, Italy) following the manufacturer’s instruction. The kit is a rapid semiquantitative dot-ELISA-based system licensed to determine the antibody titers against CPV-2, CDV, and CAdV-1. The test has been previously validated, showing good values of specificity and sensitivity for each pathogen and can be applied in practice, as indicated in the WSAVA guidelines and in many other studies [1,2,34,43,44,45,46].

In this rapid test, the antibody concentration is defined by the color intensity of the resulting spots compared with the “S” units on a scale from 1 to 6. An S value of 0 (S0) was standardized by the manufacturer as being equivalent to an antibody titer of <1:20 for CPV-2, <1:8 for CDV, and <1:4 for CAdV-1. An S value of 3 (S3) was standardized by the manufacturer to be the equivalent of 1:80 for CPV-2, 1:32 for CDV, and 1:16 for CAdV-1. Following previous studies that validated the kit for the detection of antibodies against CPV-2, CDV, and CAdV-1, antibody titers equal to or higher than S3 values were considered indicative of a significant positive response, representing the specific protection against these three diseases (Table S1). Results were divided into four categories (unprotected, weak positive, medium positive, and high positive) based on the threshold values of each pathogen; dogs with antibody titers equal to the threshold value were considered medium positive. Medium to high positive results were expressed as Protective Antibody Titers (PATs) (Table S2).

### 2.3. Statistical Analysis

Statistical analyses were performed using GraphPad Prism 9 (La Jolla, CA, USA) considering statistically significant values at *p* < 0.05. The Chi-square (χ^2^) analysis was used to determine significant differences between protected and unprotected vaccinated dogs. Antibody titer data were transformed with log_2_. The Shapiro–Wilk test was used to verify the normal distribution of data, and not-parametric Kruskal–Wallis and Mann–Whitney tests were also used.

## 3. Results

### 3.1. Dog Population

A total of 1,027 canine serum/plasma samples were included in the study. Of these, 966 were owned dogs (94.0%) and 61 were shelter dogs (6.0%). Of the 1,027 dogs analyzed in this study, 560 (56%) were females (392 sexually intact and 168 neutered) and 467 (44%) were males (395 sexually intact and 72 neutered). Collectively, 787 dogs (76.6%) were entire while 240 (23.4%) were neutered. The age ranged from 4 months to 19 years, with 129 being puppies (12.6%), 531 adults (51.7%), 271 seniors (26.4), and 96 geriatrics (9.3). Considering the breed, 695 dogs were of pure breed (67.7%), while 332 were crossbred (32.3%); the most representative breeds were Labrador Retriever (89, 8.7%), followed by Golden Retriever (40, 3.9%), and German Shepherd (34, 3.3%). Considering size, 299 were small (29.1%), 360 medium (35.1%), and 368 large in size (35.8%). Furthermore, 881 dogs were healthy (85.8%), while 146 (14.2%) had one or more clinical problems that could impact immune function (above all leishmaniasis, Cushing syndrome, Addison syndrome, hypothyroidism, lymphoma, mastocytoma, and other neoplasms). Finally, 951 (92.6%) were vaccinated almost once in their life (from 1 month to 18 years before sample collection), while 76 (7.4%) were unvaccinated. Among vaccinated dogs, 489 (51.4%) were vaccinated ≤1 year before sampling, 324 (34.1%) received their last vaccination ≥1–<3 years earlier, and 115 (12.1%) were vaccinated more than 3 years earlier; for the remaining 23 dogs (2.4%), the time of last vaccination was missing due to their history (shelter dogs).

### 3.2. Antibody Titers and Protection of the Entire Cohort

Specific antibody titers ranged from <1:20 to >1:640 for CPV-2, from <1:8 to >1:256 for CDV, and from <1:4 to >1:128 for CAdV-1. Specific PATs for CPV-2, CDV, and CAdV-1 were observed in 933 (90.8%), 705 (68.6%), and 820 (79.8%) dogs of the entire population of 1,027 animals, respectively. These percentages were even higher considering only the 951 vaccinated dogs (93.0%, 79.0%, and 81.8%, respectively). The distribution of PATs in the three dog populations considered (all 1027 dogs, 951 vaccinated dogs, and 76 unvaccinated dogs) divided into categories (sex and reproductive status, age, size, health status, and time elapsed since the last vaccination when applicable) is shown in Table 1 and Figure S1.

**Figure 1:**
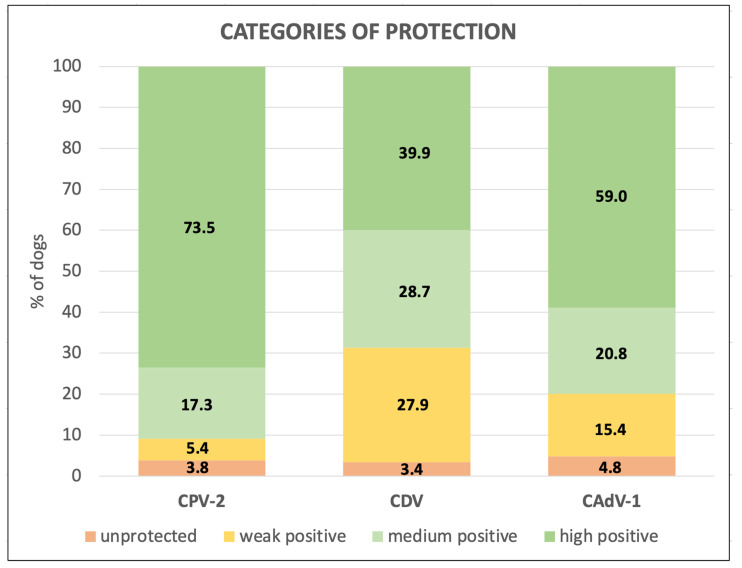
Categories of protection against Canine Parvovirus type 2 (CPV-2), Canine Distemper Virus (CDV), and Canine Adenovirus type 1 (CAdV-1) in 1027 Italian dogs.

Results were then divided into categories of protection (unprotected, weak positive, medium positive, and high positive) based on the threshold values of each pathogen (Figure 1).

**Figure 2:**
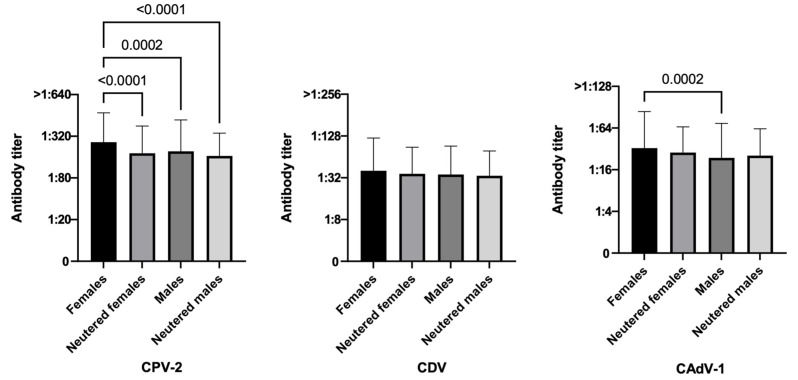
Antibody titers against Canine Parvovirus type 2 (CPV-2), Canine Distemper Virus (CDV), and Canine Adenovirus type 1 (CAdV-1), considering the variable sex and reproductive status of the 951 vaccinated dogs (Kruskal–Wallis test).

**Figure 3:**
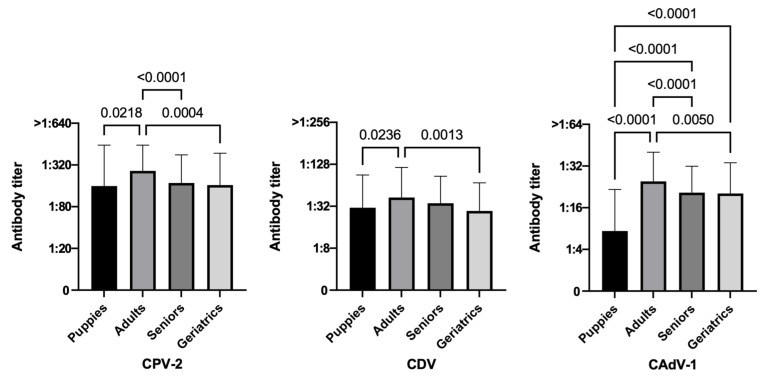
Antibody titers against Canine Parvovirus type 2 (CPV-2), Canine Distemper Virus (CDV), and Canine Adenovirus type 1 (CAdV-1), considering the variable age of the 951 vaccinated dogs (Kruskal–Wallis test).

**Figure 4:**
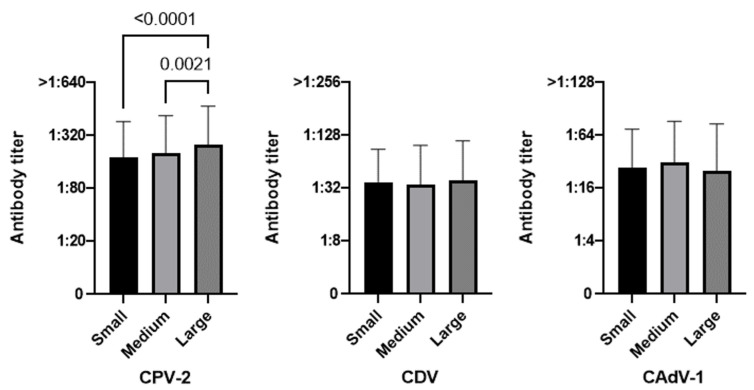
Antibody titers against Canine Parvovirus type 2 (CPV-2), Canine Distemper Virus (CDV), and Canine Adenovirus type 1 (CAdV-1), considering the variable size of the 951 vaccinated dogs (Kruskal–Wallis test).

**Figure 5:**
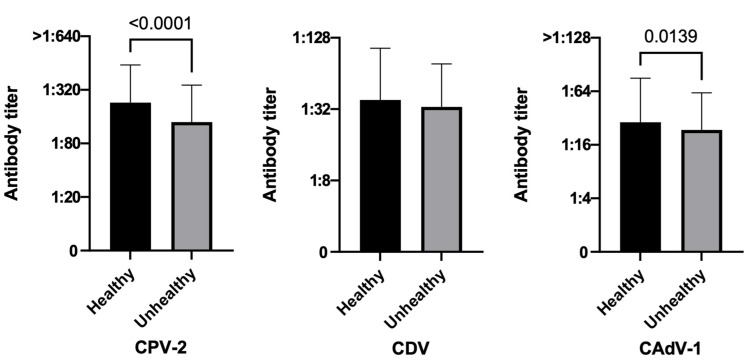
Antibody titers against Canine Parvovirus type 2 (CPV-2), Canine Distemper Virus (CDV), and Canine Adenovirus type 1 (CAdV-1), considering the variable health status of the 951 vaccinated dogs (Mann–Whitney test).

**Figure 6:**
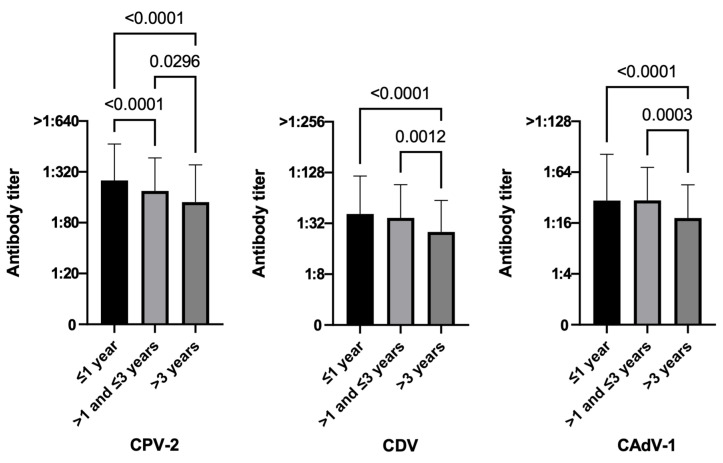
Antibody titers against Canine Parvovirus type 2 (CPV-2), Canine Distemper Virus (CDV), and Canine Adenovirus type 1 (CAdV-1), considering the time from the last vaccination of the 951 vaccinated dogs (Kruskal–Wallis test).

Table 2 refers to the results of the Chi square test applied to 951 vaccinated dogs, and Figure 2, Figure 3, Figure 4, Figure 5 and Figure 6 show the statistical results related to the 951 vaccinated dogs (see below).

In Table 3, the 90 dogs (8.8%) deemed seronegative for one or more virus antigens are reported.

Half of the entire cohort of 1,027 dogs (50.6%, 520 dogs) had a good protection (PATs equal or higher than the threshold values) for all three infectious agents. This percentage increased (68.5%, 703 dogs) if titers just below the threshold values (weak positive) were also considered. Similar percentages are also achieved considering only the 951 vaccinated dogs (50% and 68.9%, respectively). Unexpectedly, more than half of the 76 unvaccinated dogs had PATs for all three diseases (64.5% CPV-2, 51.3% CDV, 55.3% CAdV-1).

After dividing the dogs positive for antibodies into categories according to the titers, for CPV-2, and to a lesser extent CAdV-1, most dogs had antibody titers higher than the threshold values (1:80 and 1:16, respectively), and were therefore well protected against these two viruses.

### 3.3. Results According to the Different Variables

#### 3.3.1. Sex and Reproductive Status

In this study, intact females had statistically higher antibody titers against CPV-2 than all other reproductive groups. Most dogs, however, were considered protected against this virus independently by their reproductive status (90–95% for all groups) (Figure 2, *p*-value < 0.0001 vs. neutered females, *p*-value < 0.0001 vs. neutered males, and *p*-value = 0.0002 vs. intact males). For CAdV-1, intact females had statistically higher antibody titers than intact males (*p*-value = 0.0002), but it was neutered dogs (females and/or males) that had higher antibody titers when compared with intact dogs (90.7% neutered females and 87.0% neutered males vs. 79% intact females and 79.9% intact males, χ^2^ *p*-value = 0.0051).

#### 3.3.2. Age

When comparing age groups, puppies tended to be the least protected age category, while adult dogs were the most protected ones for all diseases, even if for CDV the difference between adults and seniors was not statistically significant. Puppies and geriatric animals had a greater number of individuals unprotected or with low PATs for all viruses. Specifically, puppies were particularly susceptible to CAdV-1 (73.6% unprotected individuals) with statistically significant lower antibody titers than all the other groups (Figure 3, *p*-value < 0.0001 vs. adult, senior, and even geriatric groups).

#### 3.3.3. Size

When comparing breed sizes, protected and unprotected dogs were different in a statistically significant way only for CAdV-1 (Figure 4, χ^2^ *p*-value = 0.0008), probably because of the higher percentage of large dogs being unprotected (24.3%). In trying to identify how protected these dogs were, however, the only statistically significant result was for CPV-2; for this virus, in fact, large dogs had higher antibody titers than medium size (*p*-value = 0.0021) and small ones (*p*-value < 0.0001).

#### 3.3.4. Health Status

Considering the health status, 11.9% of the unhealthy group and only 6.2% of the healthy group were unprotected for CPV-2, with CPV-2 antibody titers below the threshold value of 1:80 (Figure 5, χ^2^ *p*-value = 0.0203). These results are in line with the Mann–Whitney test results (*p*-value = <0.0001) that show that the healthy group has higher antibody titers than the unhealthy one. Despite the absence of statistically significant differences for both CAdV-1 and CDV with the χ^2^ test, a statistically significant result was obtained for CAdV-1 with the Mann–Whitney test (*p*-value = 0.0139), indicating that even if both groups had similar protection rates, the healthy dogs had higher antibody titers than the unhealthy ones.

#### 3.3.5. Time Elapsed since the Last Vaccination

As more time elapses following the last vaccination, protection clearly decreased for all three viruses in terms of antibody titers (Figure 6).

For CPV-2 only, however, almost all vaccinated dogs remained protected as time passed, with antibody values at least equal to the threshold of protection. For CPV-2, all groups had statistically significant differences between them (*p*-value < 0.0001 1 year vs. >1–≤3 years, *p*-value < 0.0001 1 year vs. >3 years, *p*-value < 0.0296 >1–≤3 years vs. >3 years), while for CDV and CAdV-1, only dogs that have been vaccinated for more than 3 years hada statistically significant difference when compared with the other two groups (CDV *p*-value < 0.0001 vs. ≤1 year and *p*-value = 0.0012 vs. >1–≤3 years; CAdV-1 *p*-value < 0.0001 vs. ≤1 year and *p*-value = 0.0003 vs. >1–≤3 years). Furthermore, most dogs remained protected up to 3 years after vaccination (CPV-2 95.4%, CDV 74.4%, CAdV-1 91.4%), while the number of protected dogs begins to decrease 3 years after vaccination, although more than half of the animals always remained protected (CPV-2 87.0%, CDV 53.9%, CAdV-1 68.9%). The Chi-square test seems to sustain this trend (see Table 2, χ^2^ CPV-2 *p*-value = 0.0087, CDV *p*-value = 0.0001, CAdV-1 *p*-value < 0.0001).

Finally, 15 dogs (2 adults, 8 seniors, and 5 geriatrics; 8 small, 3 medium, and 4 large size) had been vaccinated only once in their lives when they were puppies (from 2 to 18.8 years before). Thirteen out of these fifteen dogs maintained a good protection against CPV-2 and in a lesser extent against CDV and CAdV-1 as well. None of these dogs were considered unprotected for all three core antigens.

#### 3.3.6. Breeds with High Prevalence of Non-Responder Subjects

Since Rottweiler, Doberman Pinscher and, to a lesser extent, Labrador Retriever breeds are more likely to include non-responder subjects, above all to CPV-2 (and rabies) antigens (field or vaccine ones), in this study, these breeds were also analyzed. All the 10 vaccinated Rottweiler and all the 9 vaccinated Doberman Pinscher dogs included in this study showed very high PATs (from 1:160 to >1:640) specific for CPV-2, as did the 89 vaccinated Labrador Retriever dogs, of which 82 had very high CPV-2 PATs (from ≥1:80 to >1:640), 4 were weak positive (1:40), and only 3 were unprotected (<1:20 or negative), but these last ones were three 16-week-old puppies who were completing their vaccination protocol.

## 4. Discussion

The international vaccination guidelines all agree that revaccination of adult dogs with MLV core vaccines should be applied no more frequently than every 3 years, since vaccines from any of the main international companies can stimulate a very effective long-lasting immunity in most of the vaccinated dogs.

This study provides further evidence for durable protection induced by core vaccines when dogs received the last core vaccination more than 3 years prior, and even up to more than 18 years after the last (and sometimes only) vaccination. 

The dogs in this study were mainly protected for CPV-2 in terms of both number and antibody titers. CPV-2 is ubiquitous in both urban and rural areas in Italy and abroad, and several cases of the disease are unfortunately reported every year. Its resistance in the environment is probably the major contributor to both the disease spread and the high rates of seroprotection, since CPV-2 can also infect vaccinated dogs acting as a vaccine booster. Furthermore, cats can also be infected with CPV-2 without clinical symptoms [47,48] and shed it in their feces [49,50], representing a significant source of environmental contamination for dogs. This seems to happen only rarely [51], but it should be considered in countries, such as Italy, where the number of free-roaming cats (the so-called colony cats) is quite high [52]. On the contrary, feline parvovirus (FPV), from which CPV-2 is derived, can also infect dogs, but it does not seem to be shed outside the cat [9], thus not posing a risk to the dog. In these cases, to identify the virus responsible for a positive value, it would be necessary to use PCRs that can differentiate between dog and cat viruses (CPV-2 or FPV), field and vaccine viruses, and even different canine parvovirus strains (2a, 2b or 2c) [53,54,55,56,57].

Furthermore, the high CAdV-1 rates obtained in this study may be explained by the remarkable resistance in the environment of this virus that, similar to CPV-2, can act as natural booster in dogs that come into contact with it. CAdV-1 infection has occasionally been described in Italy both in wild animals and in dogs [24,31,58,59,60], as elsewhere. In addition, vaccines for infectious canine hepatitis contain CAdV-2 (and not CAdV-1) that, together with *Bordetella bronchiseptica* and parainfluenza viruses, is one of the major agents of the Canine Infectious Respiratory Disease Complex (CIRDC, formerly known as kennel cough or canine infectious tracheobronchitis). Vaccines contain CAdV-2 instead of CAdV-1, the true causative agent of hepatitis, since the former CAdV-1 MLV vaccines could cause, similar to the field virus, the so-called “blue eye” (an adverse reaction due to immune complexes deposit in the uvea with neutrophil infiltration and corneal endothelial damage and oedema). This problem has been solved nowadays using, in canine hepatitis vaccines, the adenovirus type 2 instead of type 1, to which CAdV-2 is genetically and antigenically closely related [1,26,61]. CAdV-2 is spread from dog to dog through coughing and, similar to CAdV-1, can survive in the environment for a long time. It is therefore possible that after encountering this virus (for example in boarding facilities, grooming salons, dog parks, or kennels), dogs develop a good cross-immunity.

In this study, as in many others, CDV was shown to stimulate the lowest values of seroprotection. This could be related to some viral characteristics, mainly its poor environmental resistance (in this case, the possibility for dogs to acquire immunity by exposure to contaminated environments is very low) and easiness of inactivation by most common disinfectants (of the three core viruses, this is the only one with envelope), as well as its low immunogenicity and immunosuppressive potential [1,26,62]. For these reasons, PATs against CDV are lower than CPV-2 ones. It should, however, be considered that, based on its performance studies, VacciCheck considers as positive values those greater than or equal to the threshold of 1:32 [46,63], while other in-clinic tests and some laboratories that perform the gold standard tests suggest using a threshold of a 1:16. This means that dogs considered unprotected in this study with a titer of 1:16 could instead be considered protected if the threshold value was lowered to 1:16 by raising the final percentage of CDV protection to 87.9% (instead of 68.6%). Moreover, WSAVA guidelines [1] emphasize that any antibody titer detected in an adult animal is an indicator of protection because it denotes the presence of immune memory[4]. It is also true, however, that the same guidelines and many published studies state that antibody titers perfectly correlate with protection; it means that a high titer corresponds to high protection and vice versa [1,2,28,64,65,66,67]. In any case, for all the three diseases considered in this study, the majority of dogs have sufficient antibody titers to provide protection.

The results of this study confirm previous findings obtained in other studies worldwide (Table 4) reporting antibody titers measured by gold standard tests (HI, VN, IF) or by one of the commercially available in-clinic tests.

It might also be possible that apparently unprotected vaccinated dogs may instead be protected through other types of immunity. Since the core vaccines used in Italy are all MLVs (none are killed), they can, for example, stimulate strong cell-mediated and/or mucosal immunity not routinely measured [67,70]. Moreover, recent studies have challenged the dogma that immunological memory is an exclusive hallmark of the adaptive immune response, suggesting that innate immunity could also have its own memory. This “trained immunity”, linked to the non-specific effects of vaccines, appears to actively participate in defenses against reinfections, above all if MLV vaccines are used [84,85,86]. Unfortunately, similar to the cell-mediated one, this kind of immunity is not easily measurable.

Although according to many published studies sex is not significantly associated with antibody titers, in this study, intact females showed statistically higher antibody titers against CPV-2 than all other reproductive groups, while for CAdV-1, it was neutered dogs (females and/or males) that had higher antibody titers when compared with intact dogs; this means that intact females did have higher average antibody titers but it was the neutered dogs that were mostly protected against CAdV-1. The same result was reported by Lechner et al. for CDV [62], while for DiGangi et al., the most protected dogs for CAdV-1 were the males [81]; on the contrary, Lund et al. suggested that male dogs had a significantly higher risk of insufficient immunity against CAdV-1 when compared to females [76]. No statistically significant difference was found in this study for CDV when comparing dogs by sex.

In many but not in all other studies, age was significantly associated to antibody titers, but in some cases, younger dogs had higher CPV-2 PATs than older dogs [65,66,69,71,73,80], probably due to the higher number of recent vaccine administrations; in other studies, older dogs were shown to have higher CPV-2 PATs than puppies [62,72,76], probably due to a stronger immune memory. Similar results were reported for the other two pathogens, since older dogs were deemed more protected than younger ones for CDV [73,81] or CAdV-1 [76,81].

The lower protection of puppies may also be related to the well-known interference by Maternally Derived Antibodies (MDA), considered the main cause of vaccine failure in young dogs. At birth, and for their first weeks of life, puppies are protected by high titers of MDA. These begin to decrease starting from 8 to 12 weeks of age, allowing the development of active immunity, with large individual differences (related to bitch vaccination status, motherly instinct, colostrum quality, intake and absorption, newborn size and strength, timing of the intestinal barrier closure, etc.) [32,33,40,65,87,88,89,90,91,92]. These MDA are considered a double-edged sword. On the one hand, in fact, they are essential for the survival of puppies, but on the other hand, they heavily interfere with vaccination, since as long as MDA titers are high, an eventual vaccination fails. The main reason for this vaccination failure is related to neutralization of the vaccine by MDA before it succeeds in stimulating the immune system. To overcome this major problem, there is no standard vaccination protocol that fits all situations, but in order to bypass the MDA interference, all international guidelines and experts agree in proposing multiple vaccinations in puppies until at least 16 weeks of age or older [1,2,3,4,5,40,43]. MDA interference also explains why adult dogs with an incomplete first vaccination series (i.e., stop at 12 weeks of age) or with an apparently correct primary vaccination (last vaccination up to 16 weeks of age) could fail to develop antibodies [66].

On the other hand, older dogs can be more easily attacked by pathogens, and because of this increased susceptibility, a proper and continued vaccination protocol is suggested. This risk seems to increase if older dogs are not normal-weight (lean or obese) [93]. It is indeed important to remember that in the elderly, the secondary immune response (typically stimulated by a vaccine booster) is less dramatically affected by aging than the primary one (starting after the first contact with an unknown antigen) [94]. It is therefore strongly recommended that vaccinations be continued with an appropriate protocol throughout the dog’s life. This rationale will probably have to be explained to the owner who will be mistakenly convinced that his dog is too old for vaccination [35,36,40]

The larger dogs in this study were generally considered less protected than smaller ones. The size variable has rarely been investigated in studies on antibody protection. Only the study of Riedl et al. reported, as this work did, a lack of CPV-2 antibody protection significantly associated with large size (bodyweight >30 kg) [66]. Notwithstanding, dog size is known to play a role in the magnitude of the immune response, with larger dogs being less protected than smaller ones. Larger dogs are more likely to have more subcutaneous fat in the classic vaccine injection sites, whereas vaccine antigens can be sequestered and no longer be visible to the immune cells [95,96].

Considering health status, only a low percentage of the unhealthy dogs of this study were deemed unprotected for the three diseases, even if the healthy dogs always had higher antibody titers than the unhealthy ones. Killey et al. underline that there is little conclusive evidence that some diseases may affect vaccine-induced protection [67]. In the study of Mahon et al., dogs hospitalized in an intensive care unit were considered less often as seropositive to CPV-2 and CDV than expected [79]. Moreover, as the last AAHA guidelines state, if a patient with existing medical conditions or health problems is to be vaccinated, owners should sign an informed consent statement; if the dog’s immunocompetence is compromised, in fact, the risk of disease may increase [2].

The more time elapses following the last vaccination, in this study, protection clearly decreases for all three viruses in terms of antibody titers, but most dogs remain protected up to 3 years after vaccination. This result provides further evidence for a long-lasting protective immunity measurable even many years after the last vaccination (up to more than 18 years later). In addition, other studies demonstrated a large percentage of dogs maintaining sufficient PATs even three or more years after their last vaccination, above all for CPV-2 [31,65,70]. Nevertheless, since not all dogs are protected after so many years, check protection by antibody titration every 2–4 years could be the best choice to avoid immunity breakdowns.

Of the 90 dogs that were tested as seronegative, only 8 (0.8%) were negative for all three core vaccines, but six of them were unvaccinated. This should not be surprising because, as the experts always contend, vaccination is not a synonym with protection, as vaccines almost never protect 100% of the vaccinated population (neither in veterinary nor in human medicine) 100% of the time. As underlined by Killey et al. [67], there can be different reasons that may account for these seronegative results, such as waning of specific serum antibodies at the time of sampling, while maintaining a strong specific immune memory (in this case, it would be likely that dogs will seroconvert following revaccination) or concurrent medical illness. In this regard, only 9 out of the 90 seronegative dogs had a compromised health status, while the other 81 were perfectly healthy and were taken to the veterinarian for a routine health check or just for vaccination. Moreover, as already mentioned, seronegative dogs may also have mounted another type of protective immune response not so easily measurable, thus remaining healthy [67,70,84,85]. It is also possible that vaccine antigens are neutralized by existing antibodies present in vaccinated dogs at the time of vaccination before they can stimulate the immune memory of the host [28,66]. This strengthens the hypothesis that it is unlikely that a vaccine can induce a boosting effect when an animal with a specific strong immunity is revaccinated; as WSAVA guidelines state, *giving more frequent vaccines to animals in an attempt to increase antibody titers is a pointless exercise* [1]. Furthermore, due to the intrinsic characteristics of VacciCheck in term of sensitivity (ranging from 88% for CPV-2 to 94% for CAdV-1 to 100% for CDV), some negative dogs may represent false negative results, at least for CPV-2 and/or CAdV-1. In any case, in accordance with the most recent recommendations on pet vaccination, a dog seronegative for one of the three core vaccines (truly negative or false negative) should be revaccinated and then tested again to control whether seroconversion has occurred or not; if the dog fails to seroconvert, it can be a true negative dog and represent a genetic non-responder for that specific antigen [1,2,67,70]. None of the seronegative dogs of this study appears to be a non-responder, since all of the analyzed subjects of the at-risk breeds (Rottweiler, Doberman Pinscher, Labrador Retriever) showed a good antibody response for CPV-2, the pathogen toward which the response is most likely to be deficient [1,2,40].

Many of the unvaccinated dogs in this study were deemed protected for all three diseases. This peculiarity had already been reported in other works, above all in free-ranging or shelter dogs (see Table 4), indicating that some animals can acquire a natural immunity to viruses such as CPV-2 and CAdV-1 that are highly resistant in the environment, or mount a cross immunity by encountering cross-reactive pathogens, as may happen in the case of CDV. Moreover, Twark et al. [70] report a statistically significant increasing of CPV-2 titers 3 to 15 years after the dogs’ primary vaccination, demonstrating the real possibility of such an event, at least for CPV-2.

Finally, in this study, only a few dogs were deemed unprotected, above all against distemper (about 30%, which drops to 12% when considering the 1:16 titer as protective). Even if WSAVA guidelines state that any antibody titer could be a protection indicator [1], this result could mean that a good herd immunity could no longer be guaranteed for a disease that instead still represents a clinical problem worldwide, at least in some categories of dogs (shelter, stray and/or free-roaming dogs). This could prompt veterinarians to consider more frequent vaccination for this disease (every 1 to 2 years), not forgetting, however, the unavailability of monovalent distemper vaccines in many parts of the world, including Italy. Since in most European countries identification and registration of all dogs to a specific canine registry is mandatory (in Italy for more than 30 years) [97,98], the number of stray dogs is not as high as in other non-European Union countries. The dogs in this study were predominantly owned dogs and only 61 were shelter dogs, of which 26 (42.6%) were deemed unprotected against distemper, but only 16 (26.2%) considering 1:16 titer as protective (23 out of 26 were not vaccinated). 

## 5. Conclusions

This study is the first that has examined the persistence of immunity to canine core vaccines in Italian dogs, confirming that a revaccination almost always can be recommended on a three-year basis for core MLV vaccines without altering the individual’s seroprotection or even herd immunity. 

For some clinicians, abandoning the traditional annual core revaccination in favor of a 3-year interval represents a new immunization paradigm not so easy to accept. Veterinarians can be afraid of losing their clients, not considering that the 3-year interval cannot and should not be applied to other vaccinations, first and foremost leptospirosis; consequently, dogs will be vaccinated annually but with different vaccines. Secondly, veterinarians can fear leaving some animals uncovered by delaying vaccination boosters; in these cases, antibody titer tests such as VacciCheck may be very useful in monitoring canine (and feline) immunity specific for core vaccines through a careful interpretation of antibody titration results. Furthermore, this study, as others before, has identified some dogs without protective antibody titers against one or more core vaccines despite vaccination (in some cases even recent); this result is noteworthy and stresses once again the opportunity to provide regular use of the in-clinic tests to detect and monitor such dogs.

There are, however, some limitations to this study. Firstly, humoral immunity is only one part of the immune responses stimulated by vaccinations together with cell-mediated, mucosal, and even innate immunity. This means that even if a dog has a poor or no antibody titer for one or more pathogens, it does not necessarily mean it is unprotected [1,67].

Secondly, VacciCheck has an optimal sensitivity (100%) for CDV but not for CPV-2 (88%) nor for CAdV-1 (94%), creating the possibility of false-negative results for parvovirus infection and infectious canine hepatitis. On the contrary, the test has an optimal specificity (100%) for CPV-2 but not for CDV (92%) nor for CAdV-1 (93%), leading to the possibility of false-positive results for distemper and infectious canine hepatitis. Among all the in-clinic tests available on the market for this purpose, however, VacciCheck is the only one recommended by WSAVA and AAHA and the only one officially approved by regulatory authorities in USA (USDA), Canada (CFIA), Japan (MAFF), Brazil (ANVISA), and other countries [99]. Moreover, a very recent study on diagnostic accuracy of canine VacciCheck compared to gold standard tests (VN, HI) has demonstrated its reliability and usefulness in the routine screening of dogs, helping to decide if core vaccine boosters are needed [100].

Thirdly, although the large sample size of the current study is a strength of this work, it does not necessarily mean that this sample is representative of the entire canine population in Italy. It would be interesting then to continue this analysis on a larger number of subjects, thus also increasing the numerosity of the subgroups (i.e., age, sex, size, health status, and vaccination history), in addition to the origin of the dogs (owned or stray).

## Figures and Tables

**Table 1:** Percentages and numbers (*in italics* in brackets) of dogs with protective results for Canine Parvovirus type 2 (CPV-2), Canine Distemper Virus (CDV), and Canine Adenovirus type 1 (CAdV-1) according to sex and reproductive status, age, size, and health status of the whole canine population (1,027 dogs), vaccinated dogs (951 dogs, for which time elapsed since the last vaccination is also reported), and unvaccinated dogs (76 dogs).

	Protective Antibody Titers (PATs)-% (*n. of Dogs*)								
	WHOLE POPULATION (1027 Dogs)			VACCINATED DOGS (951 Dogs)			UNVACCINATED DOGS (76 Dogs)		
	CPV-2	CDV	CAdV-1	CPV-2	CDV	CAdV-1	CPV-2	CDV	CAdV-1
OVERALL VALUE	90.8 (*933/1027*)	68.6 (*705/1027*)	79.8 (*820/1027*)	93.0 (*884/951*)	70.0 *(666/951*)	81.8 (*778/951*)	64.5 (*49/76*)	51.3 (*39/76*)	55.3 (*42/76*)
Sex and reproductive status									
Intact females	91.3 (*358/392*)	65.8 (*258/392*)	77.3 (*303/392*)	94.0 (*344/366*)	67.8 (*248/366*)	79.0 (*289/366*)	53.8 (*14/26*)	38.5 (*10/26*)	53.8 (*14/26*)
Neutered females	92.3 (*155/168*)	74.4 (*125/168*)	89.3 (*150/168*)	93.2 (*150/161*)	74.5 (*120/161*)	90.7 (*146/161*)	71.4 (*5/7*)	71.4 (*5/7*)	57.1 (*4/7*)
Intact males	88.9 (*351/395*)	68.6 (*271/395*)	77.5 (*306/395*)	91.3 (*324/355*)	69.9 (*248/355*)	79.7 (*283/355*)	67.5 (*27/40*)	57.5 (*23/40*)	57.5 (*23/40*)
Neutered males	95.8 (*69/72*)	70.8 (*51/72*)	84.7 (*61/72*)	95.7 (*66/69*)	72.5 (*50/69*)	87.0 (*60/69*)	100.0 (*3/3*)	33.3 (*1/3*)	33.3 (*1/3*)
Age									
Puppies (4 months–<1 year)	74.4 (*96/129*)	53.5 (*69/129*)	31.8 (*41/129*)	79.2 (*84/106*)	54.7 (*58/106*)	26.4 (*28/106*)	52.2 (*12/23*)	47.8 (*11/23*)	56.5 (*13/23*)
Adults	96.2 (*511/531*)	74.0 (*393/531*)	90.4 (*480/531*)	98.2 (*488/497*)	75.1 (*373/497*)	92.8 (*461/497*)	67.6 (*23/34*)	58.8 (*20/34*)	55.9 (*19/34*)
Seniors	90.8 (*246/271*)	69.7 (*189/271*)	82.3 (*223/271*)	90.9 (*239/263*)	70.7 (*186/263*)	83.7 (*220/263*)	87.5 (*7/8*)	37.5 (*3/8*)	37.5 (*3/8*)
Geriatrics	83.3 (*80/96*)	56.3 (*54/96*)	79.2 (*76/96*)	85.9 (*73/85*)	57.6 (*49/85*)	81.2 (*69/85*)	63.6 (*7/11*)	45.5 (*5/11*)	63.6 (*7/11*)
Size									
Small (<10 kg)	91.0 (*272/299*)	71.9 (*215/299*)	81.9 (*245/299*)	92.4 (*267/289*)	73.7 (*213/289*)	84.1 (*243/289*)	50.0 (*5/10*)	20.0 (*2/10*)	20.0 (*2/10*)
Medium (≥10–<25 kg)	88.6 (*319/360*)	65.3 (*235/360*)	83.6 (*301/360*)	92.1 (*292/317*)	66.6 (*211/317*)	86.4 (*274/317*)	62.8 (*27/43*)	55.8 (*24/43*)	62.8 (*27/43*)
Large (≥25 kg)	92.9 (*342/368*)	69.3 (*255/368*)	74.5 (*274/368*)	94.2 (*325/345*)	70.1 (*242/345*)	75.7 (*261/345*)	73.9 (*17/23*)	56.5 (*13/23*)	56.5 (*13/23*)
Health status									
Healthy	91.4 (*805/881*)	68.1 (*600/881*)	79.5 (*700/881*)	93.8 (*758/808*)	69.4 (*561/808*)	81.6 (*659/808*)	64.4 (*47/73*)	53.4 (*39/73*)	56.2 (*41/73*)
Unhealthy	87.7 (*128/146*)	71.9 (*105/146*)	82.2 (*120/146*)	88.1 (*126/143*)	73.4 (*105/143*)	83.2 (*119/143*)	66.7 (*2/3*)	0.0 (*0/0*)	33.3 (*1/3*)
Time after vaccination *									
≤1 year	//	//	//	93.3 (*456/489*)	71.4 (*349/489*)	77.9 (*381/489*)	//	//	//
>1–≤3 years	//	//	//	95.4 (*309/324*)	74.4 (*241/324*)	91.4 (*296/324*)	//	//	//
>3 years	//	//	//	87.0 (*100/115*)	53.9 (*62/115*)	68.7 (*79/115*)	//	//	//

* This variable has been calculated in 928 dogs, since for 23 vaccinated kennel dogs, the vaccination date was unknown.

**Table 2:** Percentages and numbers (*in italics* in brackets) of the Chi square test for Canine Parvovirus type 2 (CPV-2), Canine Distemper Virus (CDV), and Canine Adenovirus type 1 (CAdV-1) antibody protection according to sex and reproductive status, age, size, health status, and time elapsed since the last vaccination of the 951 vaccinated dogs.

		CPV-2			CDV			CAdV-1		
		PROTECTED		*p-*Value *	PROTECTED		*p-*Value *	PROTECTED		*p-*Value *
Statistical Variable (*Number*)		YES	NO		YES	NO		YES	NO	
**Sex**	Intact females (*366*)	94.0 (*344*)	6.0 (*22*)	0.4043	67.8 (*248*)	32.2 (*118*)	0.4479	79.0 (*289*)	21.0 (*77*)	**0.0051**
	Neutered females (*161*)	93.2 (*150*)	6.8 (*11*)		74.5 (*120*)	25.5 (*41*)		90.7 (*146*)	9.3 (*15*)	
	Intact males (*355*)	91.3 (*324*)	8.7 (*31*)		69.9 (*248*)	30.1 (*107*)		79.7 (*283*)	20.3 (*72*)	
	Neutered males (*69*)	95.7 (*66*)	4.3 (*3*)		72.5 (*50*)	27.5 (*19*)		87.0 (*60*)	13.0 (*9*)	
**Age**	Puppies (*106*)	79.2 (*84*)	20.8 (*22*)	**<0.0001**	54.7 (*58*)	45.3 (*48*)	**<0.0001**	26.4 (*28*)	73.6 (*78*)	**<0.0001**
	Adults (*497*)	98.2 (*488*)	1.8 (*9*)		75.1 (*373*)	24.9 (*124*)		92.8 (*461*)	7.2 (*36*)	
	Seniors (*263*)	90.9 (*239*)	9.1 (*24*)		70.7 (*186*)	29.3 (*77*)		83.7 (*220*)	16.3 (*43*)	
	Geriatrics (85)	85.9 (*73*)	14.1 (*12*)		57.6 (*49*)	42.4 (*36*)		81.2 (*69*)	18.8 (*16*)	
**Size**	Small (*289*)	92.4 (*267*)	7.6 (*22*)	0.5207	73.7 (*213*)	26.3 (*76*)	0.1591	83.4 (*243*)	15.6 (*46*)	**0.0008**
	Medium (*317*)	92.1 (*292*)	7.9 (*25*)		66.6 (*211*)	33.4 (*106*)		86.4 (*274*)	13.6 (*43*)	
	Large (*345*)	94.2 (*325*)	5.8 (*20*)		70.1 (*242*)	29.9 (*103*)		75.7 (*261*)	24.3 (*84*)	
**Health status**	Healthy (*808*)	93.8 (*758*)	6.2 (*50*)	**0.0203**	69.4 (*561*)	30.6 (*247*)	0.3733	81.6 (*659*)	18.4 (*149*)	0.7245
	Unhealthy (*143*)	88.1 (*126*)	11.9 (*17*)		73.4 (*105*)	26.6 (*38*)		83.2 (*119*)	16.8 (*24*)	
**Time after vaccination**	≤1 year (*489*)	93.3 (*456*)	6.7 (*33*)	**0.0087**	71.4 (*349*)	28.6 (*140*)	**0.0001**	77.9 (*381*)	22.1 (*108*)	**<0.0001**
	>1-≤3 years (*324*)	95.4 (*309*)	4.6 (*15*)		74.4 (*241*)	25.6 (*83*)		91.4 (*296*)	8.6 (*28*)	
	>3 years (*115*)	87.0 (*100*)	13.0 (*15*)		53.9 (*62*)	46.1 (*53*)		68.9 (*79*)	31.3 (*36*)	

* **in bold**, statistically significant *p*-values.

**Table 3:** Percentages (*numbers and health status*) of dogs showing as negative to at least one virus, i.e. Canine Parvovirus type 2 (CPV-2), Canine Distemper Virus (CDV), and Canine Adenovirus type 1 (CAdV-1).

NEGATIVE DOGS	WHOLE POPULATION (1027 Dogs) % (*Number & Health*)	VACCINATED DOGS (951 Dogs) % (*Number & Health*)	UNVACCINATED DOGS (76 Dogs) % (*Number & Health*)
Only for CPV-2	1.7 (*17–15H* + *2U*)	1.6 (*15*–*13H* + *2U*)	2.6 (*2*–*2H*)
Only for CDV	1.8 (*18*–*17H* + *1U*)	1.6 (*15*–*14H* + *1U*)	3.9 (*3*–*3H*)
Only for CAdV-1	2.7 (*28*–*25H* + *3U*)	2.6 (*25*–*22H* + *3U*)	3.9 (*3*–*3H*)
For CPV-2 and CDV	0.5 (*5*–*3H* + *2U*)	0.2 (*2*–*2U*)	3.9 (3–3H)
For CPV-2 and CAdV-1	1.0 (*10*–*10H*)	0.4 (*4*–*4H*)	7.9 (*6*–*6H*)
For CDV and CAdV-1	0.4 (*4*–*3H* + *1U*)	0.3 (*3*–*2H* + *1U*)	1.3 (*1*–*1H*)
For CPV-2, CDV and CAdV-1	0.8 (*8*–*8H*)	0.2 (*2*–*2H*)	7.9 (6*–*6H)
**Total**	8.8 (*90*–*81H + 9U*)	6.9 (*66*–*57H + 9U*)	31.6 (*24*–*24H*)

***H*** = *healthy*–***U*** = *Unhealthy*.

**Table 4:** Percentages of protective titers against Canine Parvovirus type 2 (CPV-2), Canine Distemper Virus (CDV), and Canine Adenovirus type 1 (CAdV-1) detected in other studies on specific antibody titration in dogs worldwide by means of gold standard tests or in-clinics test kits.

				% of Protection		
Authors (Year)	[Reference]	Country	No. Dogs	CPV-2	CDV	CAdV-1
Tennant et al. (1991)	[68]	UK	190	70.0	84.0	//
McCaw et al. (1998)	[69]	USA	122	73.0	79.0	//
Twark et al. (2000)	[70]	USA and Canada	1441	95.1	97.6	//
Bohm et al. (2004)	[31]	UK	144	95.0	71.5	82.0
Mouzin et al. (2004)	[30]	USA and Canada	322	98.1	98.1	98.4
Ottiger et al. (2006)	[71]	Switzerland and Germany	260	64.0	83.0	//
Lechner et al. (2010)	[62]	USA	431 *	67.0	43.2	//
Adam et al. (2011)	[72]	Trinidad and Tobago	92 *	78.3	39.1	//
Taguchi et al. (2011)	[73]	Japan	1031	86.0	72.0	71.0
Mitchell et al. (2012)	[28]	Australia	235	97.4	84.1	95.7
Litster et al. (2012)	[74]	USA	102	97.9	93.8	//
Belsare et al. (2013)	[75]	India	77	88.0	73.0	68.0
Lund et al. (2013)	[76]	Denmark	322	88.5	87.3	85.1
Belsare et al. (2014)	[77]	India	219 *	88.0	72.0	77.0
Riedl et al. (2015)	[66]	Germany	100	86.0	//	//
Curi et al. (2016)	[78]	Brazil	320 *	97.0	15.0	27.8
Mahon et al. (2017)	[79]	USA	80	81.0	50.0	//
Killey et al. (2018)	[67]	UK	486	98.5	95.7	97.3
den Besten (2018)	[65]	Netherland	929	97.5	90.7	91.6
Kim et al. (2018)	[80]	Korea	78	100	94.8	//
DiGangi et al. (2019)	[81]	Ecuador	154	95.0	66.0	60.0
Home et al. (2022)	[82]	India	97 *	100.0	54.0	66.0
Sadauda et al. (2022)	[83]	Nepal	163 *	17.0	33.0	//
This study (2023)	//	Italy	1027	90.8	68.6	79.8

* Unvaccinated free-ranging/shelter dogs.

## Data Availability

The authors confirm that the datasets analyzed during the study are available from the first author or the corresponding author upon reasonable request.

## Supplementary Materials

The following supporting information can be downloaded at https://www.mdpi.com/article/10.3390/life13020587/s1, Table S1: VacciCheck: correspondence between S scale units and antibody titers, sensitivity, and specificity for Canine Parvovirus type 2 (CPV-2), Canine Distemper Virus (CDV), and Canine Adenovirus type 1 (CAdV-1); Table S2: Classification of protection categories for Canine Parvovirus type 2 (CPV-2), Canine Distemper Virus (CDV), and Canine Adenovirus type 1 (CAdV-1) based on antibody titers of VacciCheck. Figure S1: Specific antibody titers for Canine Parvovirus type 2 (CPV-2), Canine Distemper Virus (CDV), and Canine Adenovirus type 1 (CAdV-1) in 1027 Italian dogs.
