# Supplementary material for: Prevalence of Serum Antibody Titers against Core Vaccine Antigens in Italian Dogs
Source: Life (Basel). 2023 Feb 20;13(2):587. doi: 10.3390/life13020587 (PMC9961557; doi:10.3390/life13020587)
Supplement: Supplementary file 1 [file life-13-00587-s001.zip › life-2195311-supplementary.pdf]

Table S1. VacciCheck: correspondence between S scale units and antibody titers, sensitivity, and specificity for Canine Parvovirus type 2 (CPV-2), Canine Distemper Virus (CDV), and Canine Adenovirus type 1 (CAdV-1)

	**CPV-2 (%)**	**CDV (%)**	**CAdV-1 (%)**
S0	<1:20	<1:8	<1:4
S1	1:20	1:8	1:4
S2	1:40	1:16	1:8
**S3 (threshold)**	**1:80**	**1:32**	**1:16**
S4	1:160	1:64	1:32
S5	1:320	1:128	1:64
S6	1:640	1:256	1:128
>S6	>1:640	>1:256	>1:128
Sensitivity (%)	*88*	*100*	*94*
Specificity (%)	*100*	*92*	*93*

Table S2. Classification of protection categories for Canine Parvovirus type 2 (CPV-2), Canine Distemper Virus (CDV), and Canine Adenovirus type 1 (CAdV-1) based on antibody titers of VacciCheck

**Categories**	**CPV-2**	**CDV**	**CAdV-1**
Non protected	<1:20	<1:8	<1:4
Weak Positive	≥1:20 to <1:80	≥1:8 to <1:32	≥1:4 to <1:16
Medium Positive	≥1:80*-<1:160	≥1:32*-<1:64	≥1:16*-<1:32
High Positive	≥1:160	≥1:64	≥1:32

* Titers with asterisk represent threshold values

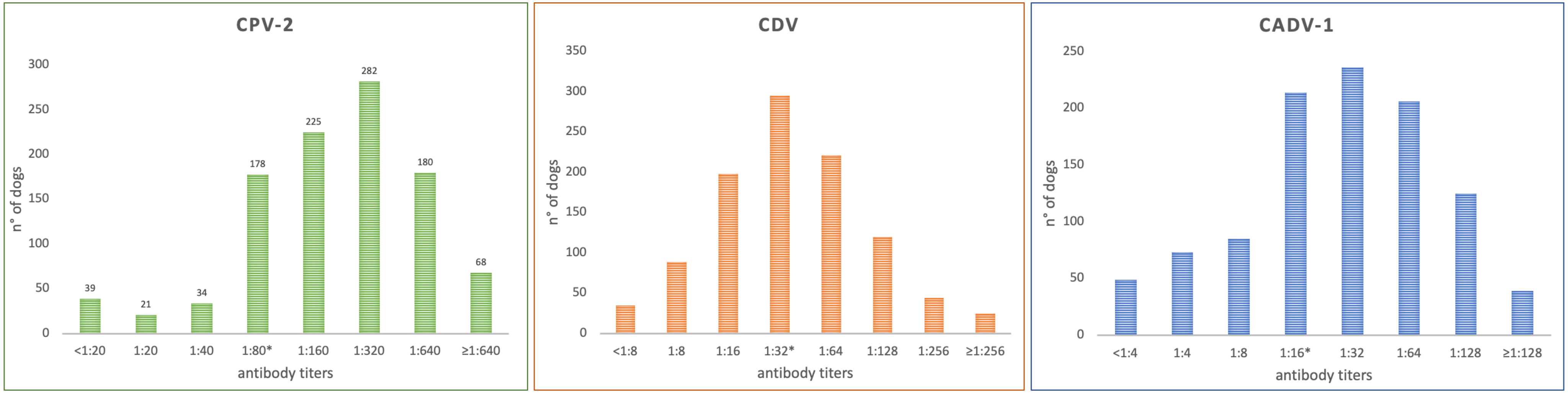

Figure S1. Specific antibody titers for Canine Parvovirus type 2 (CPV-2), Canine Distemper Virus (CDV), and Canine Adenovirus type 1 (CAdV-1) in the 1,027 Italian dogs (titers with asterisk (*) represent the threshold values)
